# A Clinical Approach for Defining the Threshold between Low and Medium Anti-Cardiolipin Antibody Levels for QUANTA Flash Assays

**DOI:** 10.3390/antib5020014

**Published:** 2016-05-25

**Authors:** Gabriella Lakos, Chelsea Bentow, Michael Mahler

**Affiliations:** Inova Diagnostics, Inc., 9900 Old Grove Road, San Diego, CA 92131-1638, USA; cbentow@inovadx.com (C.B.); mmahler@inovadx.com (M.M.)

**Keywords:** antiphospholipid syndrome, anticardiolipin antibodies, low/medium antibody threshold, chemiluminescent immunoassay

## Abstract

The threshold between low and medium antibody levels for anticardiolipin (aCL) and anti-β2 glycoprotein I antibodies (aβ2GPI) for the diagnosis of antiphospholipid syndrome (APS) remains a matter of discussion. Our goal was to create a protocol for determining the low/medium antibody cut-off for aCL antibody methods based on a clinical approach, and utilize it to establish the clinically-relevant low/medium threshold for QUANTA Flash aCL chemiluminescent immunoassay (CIA) results. The study included 288 samples from patients with primary APS (*n* = 70), secondary APS (*n* = 42), suspected APS (*n* = 36), systemic lupus erythematosus (SLE) without APS (*n* = 96) and other connective tissue diseases (*n* = 44). All samples were tested for IgG and IgM aCL antibodies with QUANTA Flash CIA, along with traditional enzyme-linked immunosorbent assays (ELISAs) (QUANTA Lite). The assay specific low/medium threshold for QUANTA Flash aCL IgG and IgM assays (*i.e.*, the equivalent of 40 GPL and MPL units) was established as 95 and 31 chemiluminescent units (CU), respectively, based on clinical performance and comparison to QUANTA Lite ELISAs. Agreement between CIA and ELISA assay results improved substantially when the platform-specific low/medium antibody threshold was used, as compared to agreement obtained on results generated with the assay cutoff: Cohen’s *kappa* increased from 0.85 to 0.91 for IgG aCL, and from 0.59 to 0.75 for IgM aCL results. This study describes a clinical approach for establishing the low/medium antibody threshold for aPL antibody assays, and successfully employs it to define 95 and 31 CU, respectively, as the low/medium cut point for QUANTA Flash aCL IgG and IgM results. This study can serve as a model for labs wishing to establish the appropriate low/medium aPL antibody threshold when implementing new aPL antibody assays.

## 1. Introduction

The updated classification criteria for definite antiphospholipid syndrome (APS), also known as Hughes syndrome, specifies anticardiolipin (aCL) and anti-β_2_-glycoprotein I (β_2_GPI) antibodies of IgG and/or IgM isotype in medium or high titer as one of the laboratory criteria [1]. As inter-laboratory agreement between aCL measurements is known to be poor due to inconsistencies of the cut-off, calibration, and other methodological issues [2,3,4], the committee recommends reporting positive results in ranges of positivity (*i.e.*, low-medium-high) to achieve better inter-run and inter-laboratory agreement than that obtained with quantitative results only [1,5]. For aCL antibodies measured by enzyme-linked immunosorbent assays (ELISA), the international consensus states that values above 40 IgG and IgM Phospholipid (GPL or MPL) units, or above the 99th percentile of the values obtained on reference subjects are considered medium or high titer aCL antibodies. The committee overseeing the revised classification criteria has acknowledged the lack of suitable evidence on this issue, but stated that these values should be used “until international consensus is reached” [1].

This concept, however, has several shortcomings. First, the 99th percentile often defines values which are significantly different from the recommended 40 GPL or MPL units [5]. In fact, the value depends on the performance characteristics of the particular assay, the statistical method, and the reference population that is used to establish the cut-off. Additionally, in the absence of a reference method, and in the light of the analytical diversity of aPL antibody assays, the use of the same unit type (GPL and MPL) by itself is not sufficient to achieve harmonization between antiphospholipid (aPL) antibody assays. This is evident by the different cut-off values of different brands of kits, and the wide range of results reported by labs during proficiency testing surveys [2,3,4,5]. Differences exist not only between various traditional, ELISA-based tests, but also between traditional tests and new technologies, such as chemiluminescent immunoassays (CIA) and addressable laser bead immunoassays (ALBIA) [6,7,8]. The analytical performance characteristics of these tests are often different from that of traditional technologies. Therefore, using the same low/medium threshold for all assays is unlikely to be an optimal to approach to achieve consistent and appropriate patient management.

To be able to leverage laboratory automation, aPL assays are being increasingly replaced with newer assays in the clinical lab. The switch from one method to another may be challenging for aPL antibodies, and if the change means the introduction of a different unit type, cut-off or analytical measuring range, it may create interpretation challenges. To prevent unfavorable effects on patient care, new methods should be carefully evaluated, compared to the traditional methods, and potential differences in unit values, unit types, and low-medium-high categories need to be analyzed and properly interpreted.

Our goal was to create and employ a protocol for the establishment of the clinically-relevant (low/medium) threshold for QUANTA Flash aCL IgG and IgM microparticle chemiluminescent immunoassays. Following the 14th International Congress on Antiphospholipid Antibodies, a committee of experts in the field of APS proposed that the threshold for aPL antibody levels should be determined using clinical approach [9], specifically, by considering the performance of a particular assay for the association with APS-related clinical symptoms. Therefore, we have set out to determine the low/medium cut-off for the QUANTA Flash aCL IgG, and IgM methods based on the clinical performance of these new tests, using traditional ELISA as reference.

## 2. Results

### 2.1. Analytical Performance

To verify the analytical performance of the QUANTA Flash IgG and IgM aCL assays, precision and linearity studies were performed. The within-run coefficients of variation (%CV) for the high and the low controls of the QUANTA Flash assays ranged from 1.0% to 3.4%. The between-day %CV ranged from 1.2% to 6.0%, and the total imprecision was between 1.5% and 6.2%. For the linearity study, results obtained on two serially-diluted samples per assay were combined in one linear regression plot. The slopes of the regression lines were 0.98 and 0.99, respectively, with coefficient of determinations (R^2^) of 1.00.

### 2.2. Threshold between Low and Medium Antibody Levels

**Figure 1:**
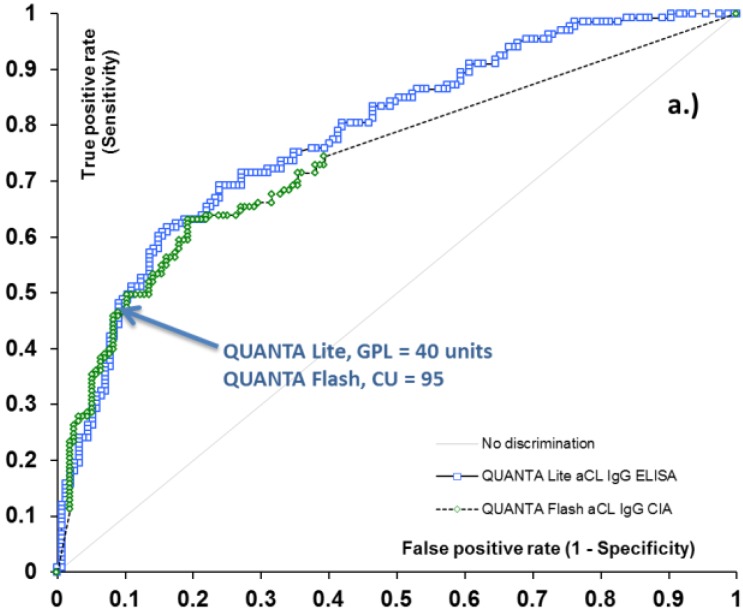

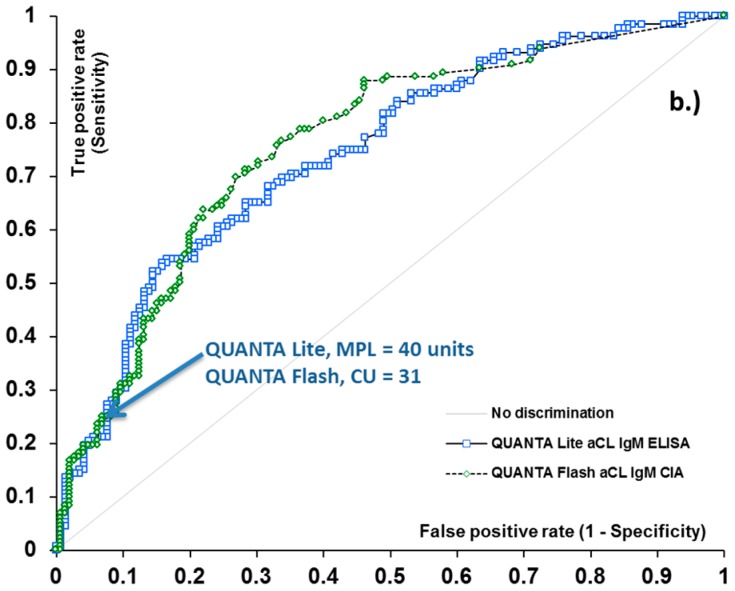
Receiver operating characteristic (ROC) analysis of aCL IgG (**a**) and IgM (**b**) methods for antiphospholipid syndrome (APS)-related clinical symptoms. Arrows indicate the clinically relevant threshold between low and medium titer for aCL assays.

40 GPL and MPL have previously been suggested as the thresholds between low and medium-high aCL antibody levels. To verify the relevance of this value as a clinically significant antibody titer, we determined the clinical sensitivity and specificity of the QUANTA Lite aCL ELISA assays for APS-related clinical symptoms (venous thrombosis, arterial thrombosis, and obstetric complications) at the 40 GPL and MPL level. At this threshold, the sensitivity of the aCL IgG and IgM ELISA was 48.1% and 25.0%, with 91.0% and 92.4% specificity, respectively (Table 1). These values indicate that at 40 GPL and MPL cut-off, the aCL ELISAs indeed deliver clinically-relevant results. Next, we performed receiver operating characteristic (ROC) analysis on QUANTA Flash aCL IgG, and IgM results to calculate the threshold that provides the same or similar clinical performance (sensitivity and specificity) (Figure 1). We were able to identify CU thresholds where the clinical sensitivity of the QUANTA Flash aCL IgG and IgM assays was essentially identical to that of the QUANTA Lite tests at the 40 unit threshold. The associated specificity values were also the same as those for QUANTA Lite. These threshold values were determined to be 31 CU for aCL IgM and 95 CU for aCL IgG (Table 1). These data points can be identified on the ROC curves as a point where the two curves cross each other (Figure 1). These results indicate that QUANTA Flash aCL assays deliver similar clinical performance at 95 and 31 CU threshold (for IgG and IgM, respectively) as that of the QUANTA Lite assays at the 40 GPL and MPL cut-off; in other words, the results suggest the equivalency of the QUANTA Flash 95 and 31 CU with the conventional 40 GPL and MPL low/medium threshold commonly utilized in traditional assays.

### 2.3. Qualitative Agreement and Quantitative Correlation between Methods

ROC curve analysis resulted in area under the curve (AUC) values ranging from 0.72 to 0.78 for ELISA and CIA methods, and demonstrated very similar diagnostic performance for the two platforms (Figure 1). Additionally, good qualitative agreement was found between CIA and ELISA methods, with overall agreements ranging from 84.9% (aCL IgM assays) to 93.1% (aCL IgG assays). Cohen’s kappa coefficients were 0.59 and 0.85, implying moderate to substantial agreement. Quantitative results also showed significant correlation between the methods, with Spearman’s rho of 0.74 and 0.83 (*p* < 0.0001 for both) (Table 2). The analysis of discrepant results revealed that the majority of these samples had low positive (*i.e.*, clinically less significant) antibody levels. Indeed, when the platform-specific threshold between low and medium positive samples (40 GPL and 40 MPL units for aCL ELISAs, and 95 CU and 31 CU for QUANTA Flash aCL IgG and IgM assays) was used as the cut-off, the agreement between the platforms substantially improved, with total agreement reaching 96.5% and kappa of 0.91 for aCL IgG assays, and total agreement of 93.5% and kappa of 0.75 for aCL IgM assays (Table 2).

## 3. Discussion

This study describes an experimental protocol for determining the low/medium antibody threshold for aPL antibody methods. Using this approach, we have identified 95 and 31 CU as low/medium threshold for results generated with the QUANTA Flash aCL IgG and IgM assays, respectively.

Although the association between thrombotic complications and antiphospholipid antibodies was first demonstrated more than 30 years ago [10], APS still poses diagnostic challenges in routine clinical practice. The characteristic clinical symptoms of the disease are actually more frequently present in non-APS than in APS patients, and the hallmark antibodies of the syndrome can occur as natural or infection-induced antibodies [1]. Rigorous specification of the clinical symptoms and laboratory results promotes accurate diagnosis [1]; however, the analytical diversity and less than optimal reproducibility of aPL results continue to make interpretation of aPL antibody results challenging. Defining the threshold between low (*i.e.*, clinically less significant) and medium-high (clinically more significant) aCL and β2GPI antibody levels helps distinguish APS patients from other diseases, but may lead to inappropriate decisions if interpreted improperly. In spite of continuous harmonization efforts [11,12], inter-laboratory portability of aPL results remains suboptimal [2,3,4], and the emergence of new platforms and technologies are bringing additional analytical variability and potential confusion into the measurement process.

The QUANTA Flash aCL and β2GPI tests are microparticle-based chemiluminescent immunoassays. Although the clinical performance of these assays has been found to be good [13,14,15,16], the wide AMR and the use of arbitrary chemiluminescent units have created challenges about the interpretation of the numerical unit values [14,17]. In particular, the lack of definition for low/medium antibody threshold hinders diagnostic efforts. Mathematical conversion of CU values to GPL and MPL units was found to be impractical, as the CIA and ELISA assays have very different analytical and technological characteristics. Although both platforms have the same numerical cut-off (20 CU for QUANTA Flash assays and 20 GPL or MPL units for QUANTA Lite assays), the correlation between unit values is non-linear, due to the wider AMR, and the better resolution and dilution linearity of QUANTA Flash results [18]. In this study we have chosen to approach the problem from a clinical point of view, as recommended by the 14th International Congress on Antiphospholipid Antibodies Task Force [9].

We verified the clinical relevance of the 40 GPL and MPL unit threshold for the QUANTA Lite aCL assays, and determined that, at this level, the ELISAs were are able to distinguish APS patients from non-APS patients with acceptable clinical sensitivity (48.1% and 25.0%) and specificity (91% and 92.4%). Based on this performance goal, we utilized ROC analysis to identify 95 CU for IgG and 31 CU for IgM aCL antibodies as thresholds for QUANTA Flash assays providing equivalent clinical performance as that of the ELISAs at 40 GPL and MPL units. These values are, therefore, considered as the low/medium threshold for aCL antibodies measured with QUANTA Flash tests.

QUANTA Flash and QUANTA Lite aCL results showed moderate to substantial qualitative agreement (84.9% and 93.1% for IgM and IgG, respectively), and significant quantitative correlation (Spearman rho 0.74 to 0.86, *p* < 0.0001). Agreement improved significantly to 93.5% and 96.5% for IgM and IgG aCL, respectively, when the assay-specific low/medium threshold was used as the cut-off.

Establishing the low/medium antibody threshold for QUANTA Flash aCL antibody results will facilitate the utilization and help achieve correct interpretation of the results. It also ensures the continuity and consistency of patient care by using low/medium cut points that are clinically equivalent to those described in the classification criteria. In addition, as the study protocol can be utilized for any new aPL antibody test, this study can serve as a model for labs wishing to establish the appropriate low/medium aPL antibody threshold when implementing new aPL antibody assays.

## 4. Materials and Methods

### 4.1. Samples

The study included 288 samples collected at the Clinical Division of Allergy and Immunology at Jagiellonian University Medical College (Krakow, Poland) from patients referred to the clinic with the diagnosis of SLE, other systemic autoimmune disease and/or APS. The population comprised of samples from patients with primary APS (*n* = 70), secondary APS (*n* = 42) (all SLE), suspected APS patients (*n* = 36), and control sera from patients with SLE without APS (*n* = 96) and other connective tissue diseases (*n* = 44, Sjogren’s, syndrome, dermatomyositis, mixed connective tissue disease, scleroderma, undifferentiated connective tissue disease). Suspected APS patients did not completely fulfill the classification criteria, but were either aPL antibody-positive without classical clinical symptoms, or had one of the criteria clinical symptoms without medium or high levels of aPL antibody positivity. Data on the presence or absence of venous thrombosis, arterial thrombosis, and obstetric complications were available for all patients. APS diagnosis was made based on the updated Sydney APS criteria [1]. SLE patients were diagnosed according to the American College of Rheumatology criteria whenever at least four ACR criteria were fulfilled [19]. All other diagnoses were established as described before [20]. This study meets and is in compliance with all ethical standards in medicine, and informed consent was obtained from all patients according to the Declaration of Helsinki. All samples were tested for aCL antibodies using QUANTA Flash^®^ aCL (IgG, IgM) and QUANTA Lite aCL (IgG, IgM).

### 4.2. QUANTA Flash^®^ Methods

The QUANTA Flash aCL (IgG and IgM) assays (Inova Diagnostics Inc., San Diego, CA, USA) are microparticle chemiluminescent immunoassays (CIAs) that are run on the BIO-FLASH^®^ instrument (Biokit S.A., Barcelona, Spain). BIO-FLASH is a random access, rapid-response, fully-automated chemiluminescent analyzer. Results are expressed in (arbitrary) chemiluminescent units (CU). Analytical characteristics of the assays, including cut-off, measure of units and analytical measuring range (AMR) are summarized in Table 3.

### 4.3. QUANTA Lite^®^ Methods

The QUANTA Lite aCL (IgG and IgM) methods (Inova Diagnostics, San Diego, CA, USA) are traditional enzyme-linked immunosorbent assays (ELISAs) for the semi-quantitative determination of aCL antibodies in human serum. The QUANTA Lite aCL assays report results in GPL and MPL units. All QUANTA Lite ELISAs were performed according to the manufacturer’s guidelines. Analytical characteristics of the assays are summarized in Table 3. QUANTA Lite aCL assays have an equivocal range defined. For the purposes of this study, only values above the equivocal range were defined as positive.

### 4.4. Analytical Performance Assessment of QUANTA Flash Methods

Precision performance and linearity of the QUANTA Flash aCL assays were verified as part of the analytical assessment. Testing was performed according to relevant Clinical and Laboratory Standards Institute (CLSI) guidelines EP5-A2 and EP6-A. Within-run, between-days and total imprecision were determined by running two samples (the low and the high controls) in triplicate for five days. Linearity testing was performed by serially diluting two samples (one high and one low) to span the AMR for each assay, testing the dilutions in duplicate, plotting obtained values against expected values, and analyzing the results with linear regression.

### 4.5. Statistical Analyses

Data were statistically evaluated using the Analyse-it for Excel software (Version 2.30; Analyse-it Software, Ltd., Leeds, UK). Cohen’s *kappa* agreement test was used to assess concordance between portions, and Spearman’s correlation was used to evaluate quantitative relationship between unit values. Outcome was considered significant if *p* value was less than 0.05. Receiver operating characteristics (ROC) analysis was used to assess the diagnostic performance of the different immunoassays.

## 5. Conclusions

As recommended by the international committee of experts in APS, this study uses a clinical approach for establishing the low/medium antibody threshold for QUANTA Flash aCL IgG and IgM methods. This analysis will help achieve the correct interpretation of the results; moreover, it can serve as a model for labs wishing to establish the appropriate low/medium aPL antibody threshold when implementing new aPL antibody assays.

## Figures and Tables

**Table 1:** Low/medium threshold values, and associated clinical sensitivity and specificity of anticardiolipin (aCL) assays. GPL: IgG Phospholipid; CU: chemiluminescent units; MPL: IgM Phospholipid; CI: confidence interval.

Assay Characteristic	QUANTA Lite aCL IgG	QUANTA Flash aCL IgG	QUANTA Lite aCL IgM	QUANTA Flash aCL IgM
**Low/Medium** **Threshold Unit**	40 GPL	95 CU	40 MPL	31 CU
**Sensitivity, % (95% CI)** **at Threshold**	48.1 (39.4–56.9)	48.1 (39.4–56.9)	25.0 (17.9–33.3)	25.0 (17.9–33.3)
**Specificity, % (95% CI)** **at Threshold**	91.0 (85.3–95.0)	89.7 (83.8–94.0)	92.4 (86.8–96.2)	92.4 (86.8–96.2)

**Table 2:** Qualitative agreement and quantitative correlation between chemiluminescent immunoassay (CIA) and enzyme-linked immunosorbent assay (ELISA) methods at the assay-specific cut-off values and at assay-specific low/medium threshold.

QUANTA Flash CIA *vs.* QUANTA Lite ELISA				
Assay			IgG	IgM
**aCL**	**at the cut-off**	**Total % agreement (95% CI)**	93.1 (89.5–95.7)	84.9 (80.1–88.9)
		**Cohen’s kappa coefficient (95% CI)**	0.85 (0.78–0.91)	0.59 (0.48–0.70)
		**Spearman’s rho (*p*)**	0.83 (*p* < 0.0001)	0.74 (*p* < 0.0001)
	**At low/medium threshold**	**Total % agreement (95% CI)**	96.5 (93.7–98.3)	93.5 (89.9–96.1)
		**Cohen’s kappa coefficient (95% CI)**	0.91 (0.86–0.97)	0.75 (0.64–0.86)

**Table 3:** Analytical characteristics of the aCL antibody assays used in this study.

Assay	Antigen	Units of Measurement	Analytical Measuring Range	Cut-Off Value (Reference Ranges)
**QF aCL IgG**	Cardiolipin and human β2GPI	CU	2.6–2024 CU	≥20 Positive
**QF aCL IgM**	Cardiolipin and human β2GPI	CU	1.0–774 CU	≥20 Positive
**QL aCL IgG**	Cardiolipin and bovine β2GPI	GPL	0.0–150.0 GPL	<15 Negative
				15–20 Indeterminate
				>20 Positive
**QL aCL IgM**	Cardiolipin and bovine β2GPI	MPL	0.0–150.0 MPL	<12.5 Negative
				12.5–20 Indeterminate
				>20 Positive

QF = QUANTA Flash; QL = QUANTA Lite; CU = chemiluminescent units.
